# Incidentally found resectable lung cancer with the usage of artificial intelligence on chest radiographs

**DOI:** 10.1371/journal.pone.0281690

**Published:** 2023-03-10

**Authors:** Se Hyun Kwak, Eun-Kyung Kim, Myung Hyun Kim, Eun Hye Lee, Hyun Joo Shin

**Affiliations:** 1 Division of Pulmonology, Department of Internal Medicine, Allergy and Critical Care Medicine, Yongin Severance Hospital, Yonsei University College of Medicine, Yongin-si, Gyeonggi-do, Republic of Korea; 2 Department of Radiology, Research Institute of Radiological Science and Center for Clinical Imaging Data Science, Yongin Severance Hospital, Yonsei University College of Medicine, Yongin-si, Gyeonggi-do, Republic of Korea; 3 Center for Digital Health, Yongin Severance Hospital, Yonsei University College of Medicine, Yongin-si, Gyeonggi-do, Republic of Korea; Inha University Hospital, REPUBLIC OF KOREA

## Abstract

**Purpose:**

Detection of early lung cancer using chest radiograph remains challenging. We aimed to highlight the benefit of using artificial intelligence (AI) in chest radiograph with regard to its role in the unexpected detection of resectable early lung cancer.

**Materials and methods:**

Patients with pathologically proven resectable lung cancer from March 2020 to February 2022 were retrospectively analyzed. Among them, we included patients with incidentally detected resectable lung cancer. Because commercially available AI-based lesion detection software was integrated for all chest radiographs in our hospital, we reviewed the clinical process of detecting lung cancer using AI in chest radiographs.

**Results:**

Among the 75 patients with pathologically proven resectable lung cancer, 13 (17.3%) had incidentally discovered lung cancer with a median size of 2.6 cm. Eight patients underwent chest radiograph for the evaluation of extrapulmonary diseases, while five underwent radiograph in preparation of an operation or procedure concerning other body parts. All lesions were detected as nodules by the AI-based software, and the median abnormality score for the nodules was 78%. Eight patients (61.5%) consulted a pulmonologist promptly on the same day when the chest radiograph was taken and before they received the radiologist’s official report. Total and invasive sizes of the part-solid nodules were 2.3–3.3 cm and 0.75–2.2 cm, respectively.

**Conclusion:**

This study demonstrates actual cases of unexpectedly detected resectable early lung cancer using AI-based lesion detection software. Our results suggest that AI is beneficial for incidental detection of early lung cancer in chest radiographs.

## Introduction

Lung cancer is the second most commonly diagnosed cancer, with an estimated 2.2 million new cancer cases and 1.8 million deaths worldwide in 2020 [1]. It is also the leading cause of morbidity and mortality from cancer in both men and women [2]. Although the 5-year relative survival rate for lung cancer in South Korea increased from 16.6% in 2001–2005 to 34.7% in 2013–2019 [3], it still remains significantly lower than that of other cancers. This is probably because most lung cancer patients are diagnosed at an advanced stage as there are no obvious specific symptoms in the early stage of the disease [4]. The clinical stage at presentation has the greatest impact on the prognosis of lung cancer, which indicates that early detection and screening methods are of paramount importance [5].

According to the National Lung Screening Trial, which was published in 2011, lung cancer screening with annual low-dose computed tomography (CT) reduced the mortality of lung cancer by approximately 20% among those at high risk [6]. However, as screening is indicated for high-risk individuals with a smoking history of more than 30 pack-years, the current screening guidelines may miss many lung cancer patients, especially non-smokers [7, 8].

Chest radiograph is the most commonly utilized imaging modality in clinical practice; however, its use for lung cancer screening is still controversial [9]. Although there are concerns that chest radiograph may overlook malignant pulmonary nodules owing to its lower sensitivity than CT [10], some studies have also demonstrated its potential role in screening for lung cancer [11, 12]. A recent study by Koo *et al*. [13] showed that early lung cancer was detected in 38% of patients receiving chest radiographs versus 26% of those without surveillance. Furthermore, they found that surveillance with chest radiographs at intervals of less than 3 years was an independent predictor of improved survival in the women in the screened population.

In recent years, artificial intelligence (AI) was emerged as a new solution for lesion detection in medical imaging, especially for chest radiographs [14]. Many recent studies have developed and validated their own AI algorithms for chest radiographs [15, 16], which have been tested to increase the detection rate of abnormal findings, such as pulmonary nodules [17–20]. Recently, several softwares have been approved for commercial use to assist imaging interpretation in clinical practice [20–22]. However, the actual clinical adoption of AI and its usefulness have still not been verified, and many researchers have stated that proving its contribution in real-world clinical practice is necessary for the next step [21].

In the current study, we have presented actual cases of incidentally detected lung cancer in a hospital where all chest radiographs are immediately analyzed by an AI-based lesion detection software. The objective of this study was to highlight the actual benefit of AI in chest radiograph with respect to its role in the unexpected detection of resectable early lung cancer.

## Materials and methods

### Participants

The Institutional Review Board of Yongin Severance Hospital approved this retrospective study, and the requirement for informed consent was waived (IRB No. 2021-0491-001). Patients with pathologically confirmed lung cancer after surgical resection from March 2020 to February 2022 were retrospectively reviewed. Among them, the patients with incidentally detected resectable lung cancer were included in this study. We defined the incidentally detected resectable lung cancer as follows; (1) patients who had visited outpatient clinic except for pulmonology or thoracic surgery department for the evaluation or treatment of extrapulmonary diseases, (2) discovered lung nodule on initial chest radiographs by AI and confirmed it as lung cancer after the resection. We excluded patients who had initially visited the pulmonology or thoracic surgery department, were referred from another hospital owing to the suspicion of a lung abnormality in chest imaging, visited another department owing to respiratory symptoms, or underwent a health check-up or screening for lung cancer.

We retrospectively reviewed the medical records of the patients to describe the entire process of detecting lung cancer by using AI in chest radiographs. In addition to the age and sex of the included patients, information regarding the final pathologic diagnosis, tumor location, size, characteristics, TNM stage, and stage, which was based on the AJCC 8^th^ edition, were evaluated using the pathologic reports after surgical resection.

### Chest radiographs and the applied AI-based lesion detection software

All included patients had undergone chest radiograph, which was followed by CT scanning owing to suspected lung abnormalities that were observed in the radiographs. We collected the data regarding the reasons for undergoing the chest radiograph in the first place, the department that order the tests, and radiologists’ evaluation of the radiographs. Because our general hospital used a commercially available AI-based lesion detection software (Lunit INSIGHT CXR, version 2 and 3, Lunit Inc., Korea) for all chest radiographs from March 2020, we additionally assessed the AI-based results using a ResNet34-based software, which is approved for usage to analyze chest radiographs of adults [21, 23]. It was integrated into the picture archiving and communicating system (PACS) to analyze the chest radiographs to detect specific lung lesions immediately after the chest radiographs were taken.

Two versions of the software were integrated into the PACS during the study period. Version 2 was integrated for the detection of three lesions (pneumothorax, nodule, and consolidation) from March 2020 to February 2021. Lesion location was displayed in the chest radiographs by a color heatmap when the abnormality score (probability of an abnormal lesion existing) was above the preset operating point of 15%. The total abnormality score was displayed as a representative value for each radiograph by selecting the highest score for three lesions.

From March 2021 to February 2022, the upgraded version 3 was applied, and the number of detectable lesions was increased to eight (pneumothorax, nodule, consolidation, fibrosis, atelectasis, cardiomegaly, pleural effusion, and pneumoperitoneum). Additionally, this version displayed details of the detected lesion and its abnormality score separately with a grayscale contour map for localization. These two versions of software were used in different time periods and the upgraded version 3 had the function of detecting additional six lesions. Lung nodule was detected identically in these two versions and integration of different versions in two years did not affect the method of lung nodule detection.

**Fig 1:**
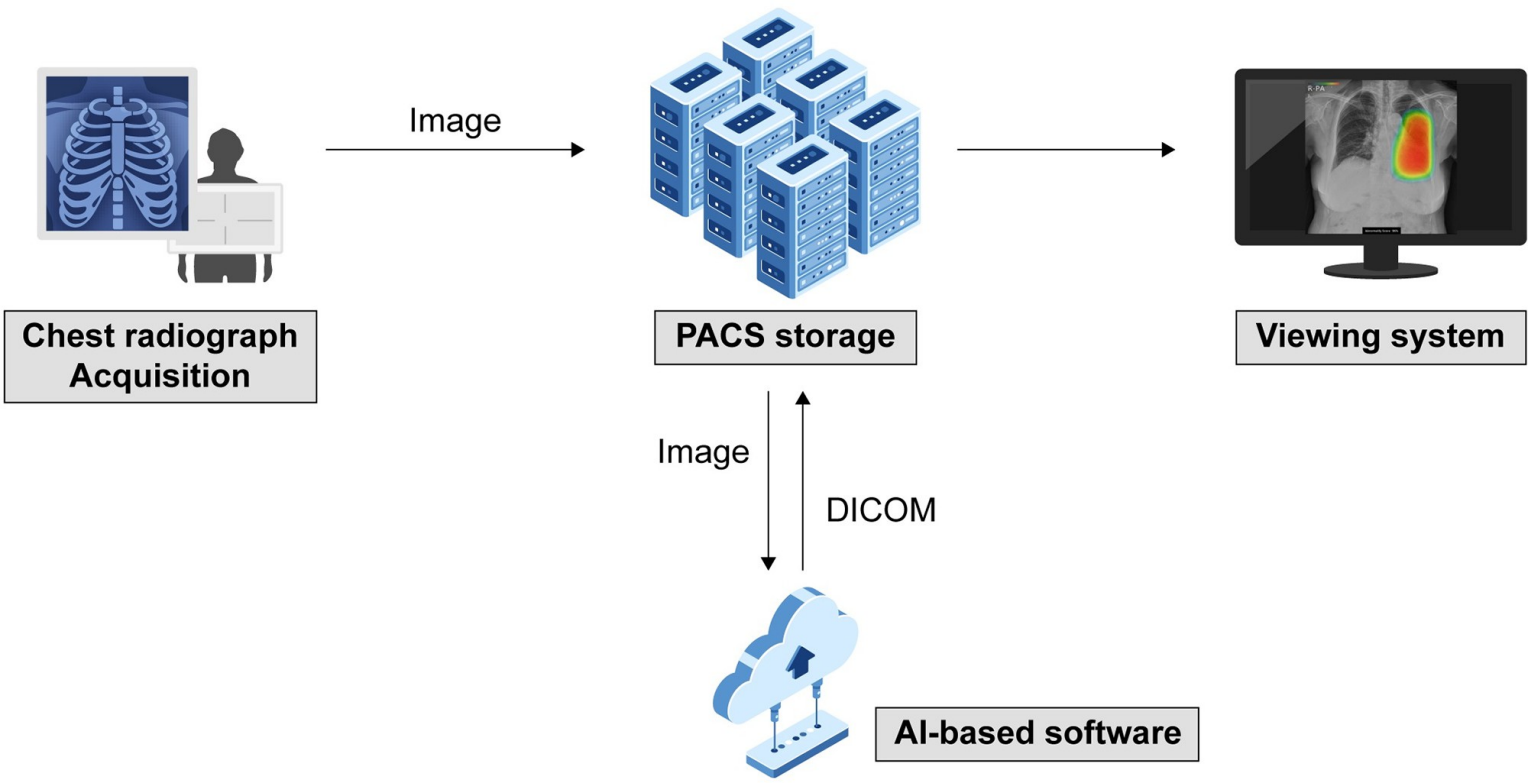
Integration of artificial intellingence results in the picture archiving and communicating system (PACS). For the included patients, we assessed the abnormality score of AI for the detected lesion in each chest radiograph. In addition, the interval between radiograph and consultation with a pulmonologist was evaluated. Whether the consultation with pulmonologists occurred before or after receiving the radiologists’ official report was assessed based on the reading time and consultation time recorded in the electronic medical records.

The software was applied to chest radiographs of all patients over 18 years old. As soon as the patient underwent chest radiograph, the image was sent to the server for processing the AI-based analyzed results. Then, the AI-based results were attached to the original radiograph as a secondary series simultaneously. Clinicians could see the AI-based results as soon as the patients underwent chest radiograph just by scrolling down to the images on PACS (Fig 1).

## Results

From March 2020 to February 2022, 75 patients were confirmed to have lung cancer on pathology following lung resection in our hospital. Among them, 13 patients (17.3%, male:female = 9:3, median = 65 years, range, 52–79 years) had incidentally detected resectable lung cancer. The remaining 62 patients were excluded because they initially visited the pulmonology or thoracic surgery department because of known lung abnormalities. Table 1 summarizes the demographic and clinical characteristics of these patients.

**Table 1:** Demographic and clinical characteristics of 13 patients.

No	Sex	Age	Reason for chest radiograph examination	Ordering Department	Smoking	AI abnormality score for the nodule (%)	Radiologist’s report	Interval between radiograph and consultation with pulmonologist (day)	Consultation before or after the radiologist’s report	Pathology	TMN	Stage^a^	Tumor location, characteristics, total size in cm (invasive size in part-solid)
1	M	52	Hypertension evaluation	Cardiology	non-smoker	43	R/O a small nodular opacity in the Rt middle lung field.	0	Before	Adeno	pT1cN0	IA3	RLL, part-solid, 2.6 (2.2)
2	M	60	Preparation for cholecystectomy	Hepatobiliary surgery	current	37	Focal nodular opacity in the Lt upper lung field, R/O granuloma or nodule.	3	After	Adeno	pT1aN0	IA1	LUL, part-solid, 2.3 (0.75)
3	M	60	Admission for MI	Cardiology	current	62	Subtle nodular opacity in the Lt upper lung field.	0	Before	Adeno	pT1N1	IIB	LUL, solid 2.1
4	M	63	Follow-up of CAOD	Cardiology	current	48	A newly visible nodular opacity in the Lt lower lung field.	0	After	SCLC^b^	pT1b/pN1 (Limited stage)	IIB	LLL, solid, 2.0
5	F	65	Preparation for knee operation	Orthopedic surgery	non-smoker	95	Mass-like opacity of about 3.7 cm in the Rt upper lung field.	7	Before	Adeno	pT2aN0	IB	RUL, solid, 3.4
6	F	65	Admission for schizophrenia	Psychiatry	ex-smoker	81	Nodular opacity of about 1 cm in the Lt middle lung field, R/O malignancy.	0	Before	Adeno	pT1b/pN0	IA2	LLL, solid, 1.9
7	M	65	Preparation for spine operation	Orthopedic	current	88	Focal consolidation or mass opacity of about 5.5 cm in the Rt upper lung field.	7	Before	Sqcc	pT3N0	IIB	RUL, solid, 5.5
8	F	71	Preparation for spine operation	Neurosurgery	non-smoker	83	Focal GGO in the Rt upper lung field.	0	After	Adeno	pT1cN0	IA3	RUL, part-solid, 3.3 (2.1)
9	M	74	Admission for ESD	Gastroenterology	ex-smoker	83	Prominent nodular opacity in the Rt upper lung field.	24	After	Adeno	pT1cN2	IIIA	RUL, solid, 2.6
10	M	75	Admission for back pain	Orthopedic surgery	non-smoker	95	Mass-like opacity of about 3 cm in the Rt lower lung field.	0	Before	Adeno	pT2aN0	IB	RML, solid, 3.4
11	M	76	Admission for stroke	Neurology	current	85	Consolidation or partial collapse in the Rt middle lung field.	0	Before	Adeno	pT2b/pN0	IIA	RUL, solid, 4.5
12	M	77	Admission for gastritis	Gastroenterology	current	50	No active lung disease.	0	Before	Sqcc	pT2/pN0	IIA	RML, solid, 2.3
13	M	79	Arrhythmia evaluation	Cardiology	ex-smoker	26	Suspicious mass-like opacity in the Rt perihilar space.	6	After	Adeno	pT1cN0	IA3	RLL, solid, 2.8

^a^Lung cancer stage according to the AJCC 8^th^ edition.

^b^This patient underwent surgery without biopsy, frozen pathology revealed that the nodule was small cell lung cancer which known to be treated concurrent chemoradiotherapy instead of surgery. Final stage was limited stage (TMN: pT1b/pN1).

Abbreviations: Adeno = adenocarcinoma, AI = artificial intelligence, CAOD = coronary artery occlusive disease, ESD = endoscopic submucosal dissection, LT = Left, MI = myocardial infarction, Rt = Right, SCLC = small cell lung cancer, Sqcc = squamous cell carcinoma.

At first, all patients were suspected to have lung abnormalities based on their initial chest radiographs, which were ordered by cardiologists (n = 4), orthopedic surgeons (n = 3), gastroenterologists (n = 2), neurologist (n = 1), neurosurgeon (n = 1), general surgeon (n = 1), and psychiatrist (n = 1). Five patients (38.5%) underwent chest radiograph before an operation or procedure involving other body parts, such as cholecystectomy, spine, or knee surgery. The remaining eight patients had undergone chest radiograph for the evaluation or treatment of extrapulmonary diseases, such as coronary artery disease, stroke, gastritis, and schizophrenia. All lesions were detected as nodules by the AI-based software, and the median abnormality score for the nodules was 78% (range, 26–95%). In eight of 13 patients (61.5%), pulmonologists were consulted for the abnormal chest radiograph findings on the same day when the chest radiographs were taken. In addition, these eight patients had received consultation before receiving the radiologist’s official report regarding the chest radiographs.

**Fig 2:**
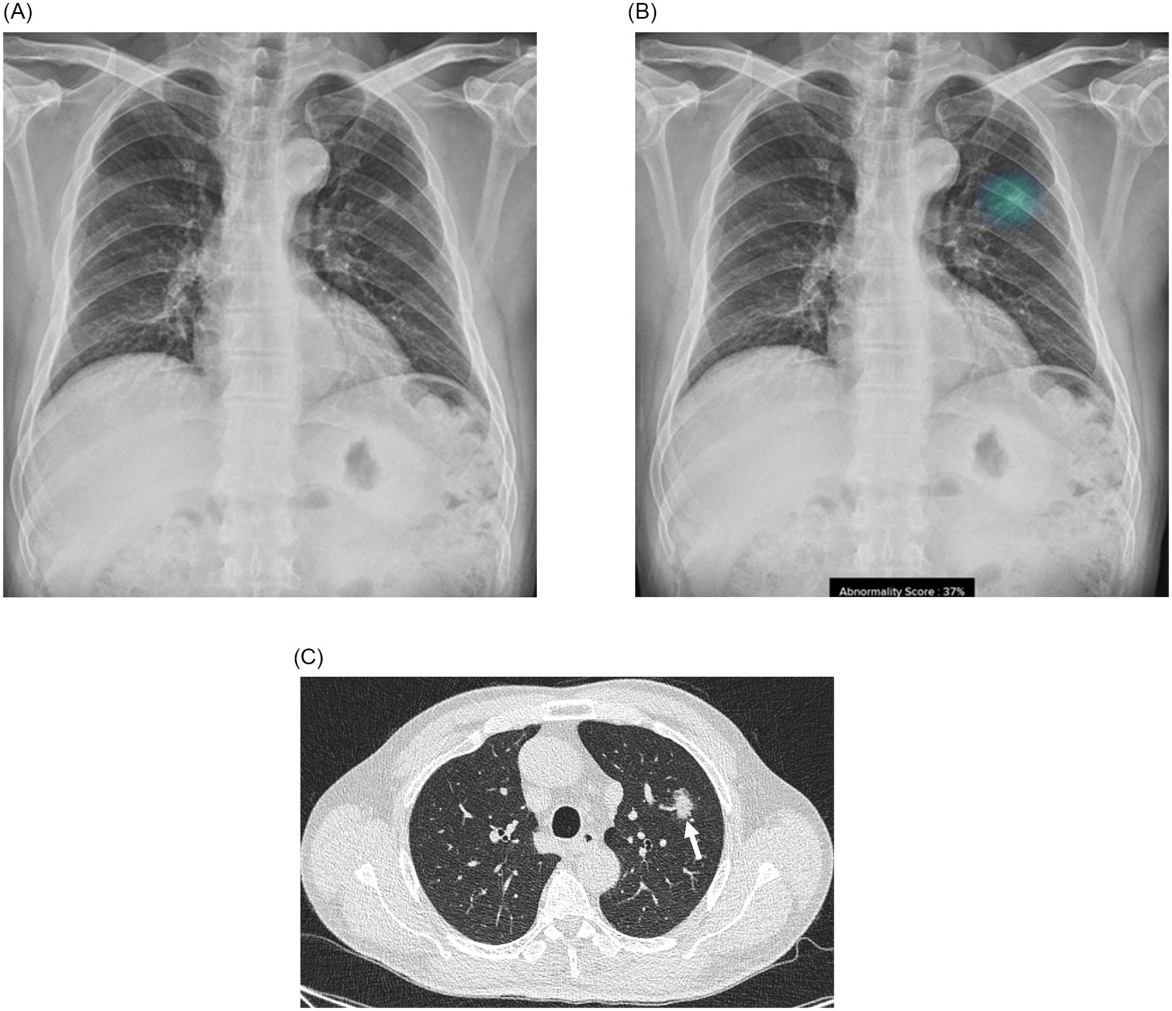
Chest radiograph and computed tomography (CT) of a 60-year-old man (#2 patient). (A) Chest radiograph was taken before performing cholecystectomy for chronic cholecystitis. (B) Artificial intelligence-based lesion detection software detected a lung nodule in the left upper lobe with an abnormality score of 37%. (C) After cholecystectomy, chest CT demonstrated a part-solid nodule in the left upper lobe (arrow), and pathology confirmed adenocarcinoma after surgical resection (total tumor size of 2.3 cm; invasive size of 0.75 cm). The final stage was IA1 (pT1aN0M0). The patient is now undergoing routine follow-up in the outpatient clinic without additional treatment.

**Fig 3:**
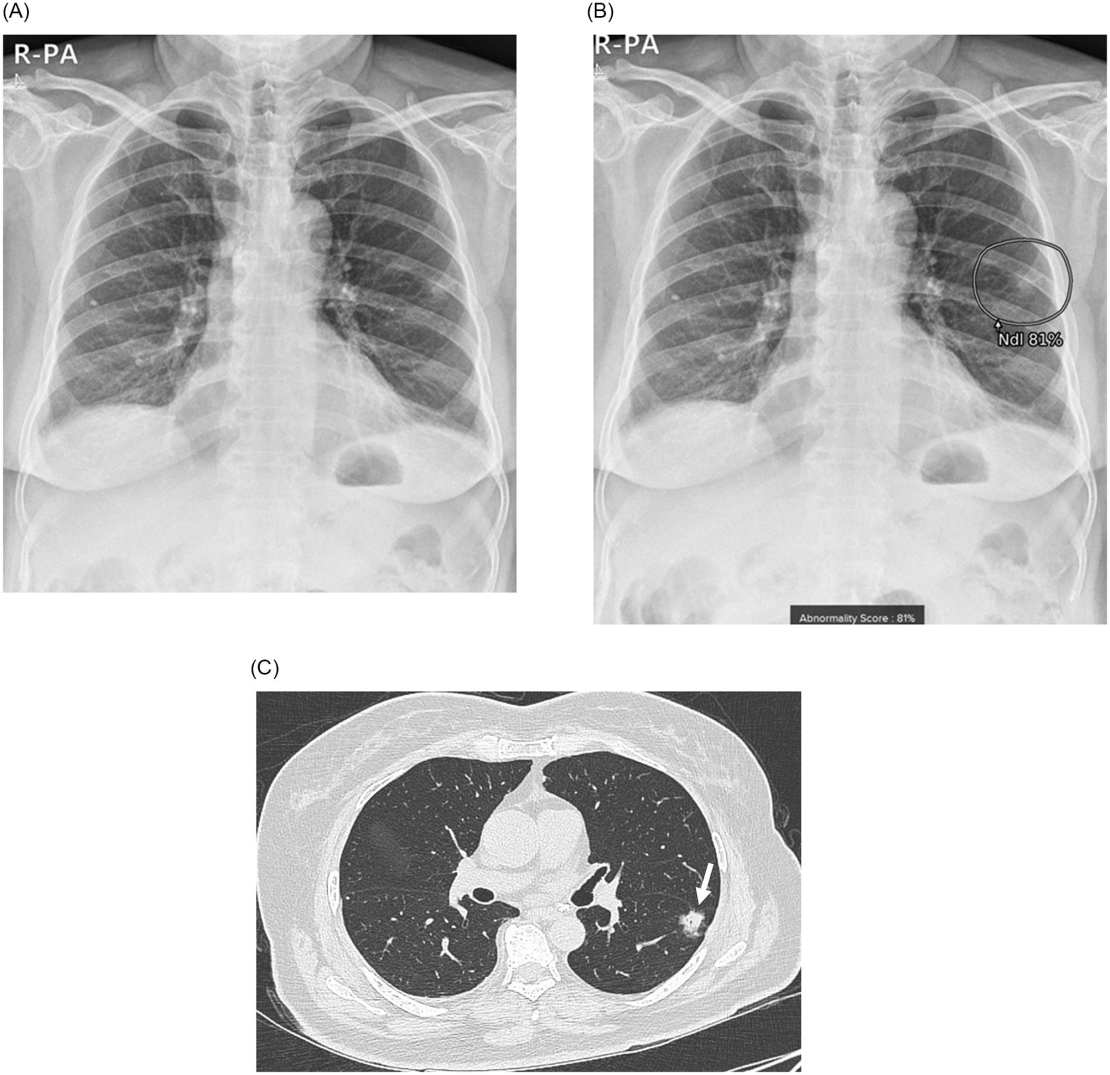
Chest radiograph and computed tomography (CT) of a 65-year-old woman (#6 patient). (A) Chest radiograph was taken at the time of admission to the psychiatric ward for schizophrenia. (B) Artificial intelligence-based lesion detection software detected a lung nodule in the left lower lung field with an abnormality score of 81%. (C) Chest CT demonstrated an irregular solid nodule in the left lower lobe (arrow), and adenocarcinoma of approximately 1.9 cm was confirmed after surgical resection. The final stage was IA2 (pT1bN0M0), and she is now receiving routine follow-up for lung cancer.

**Fig 4:**
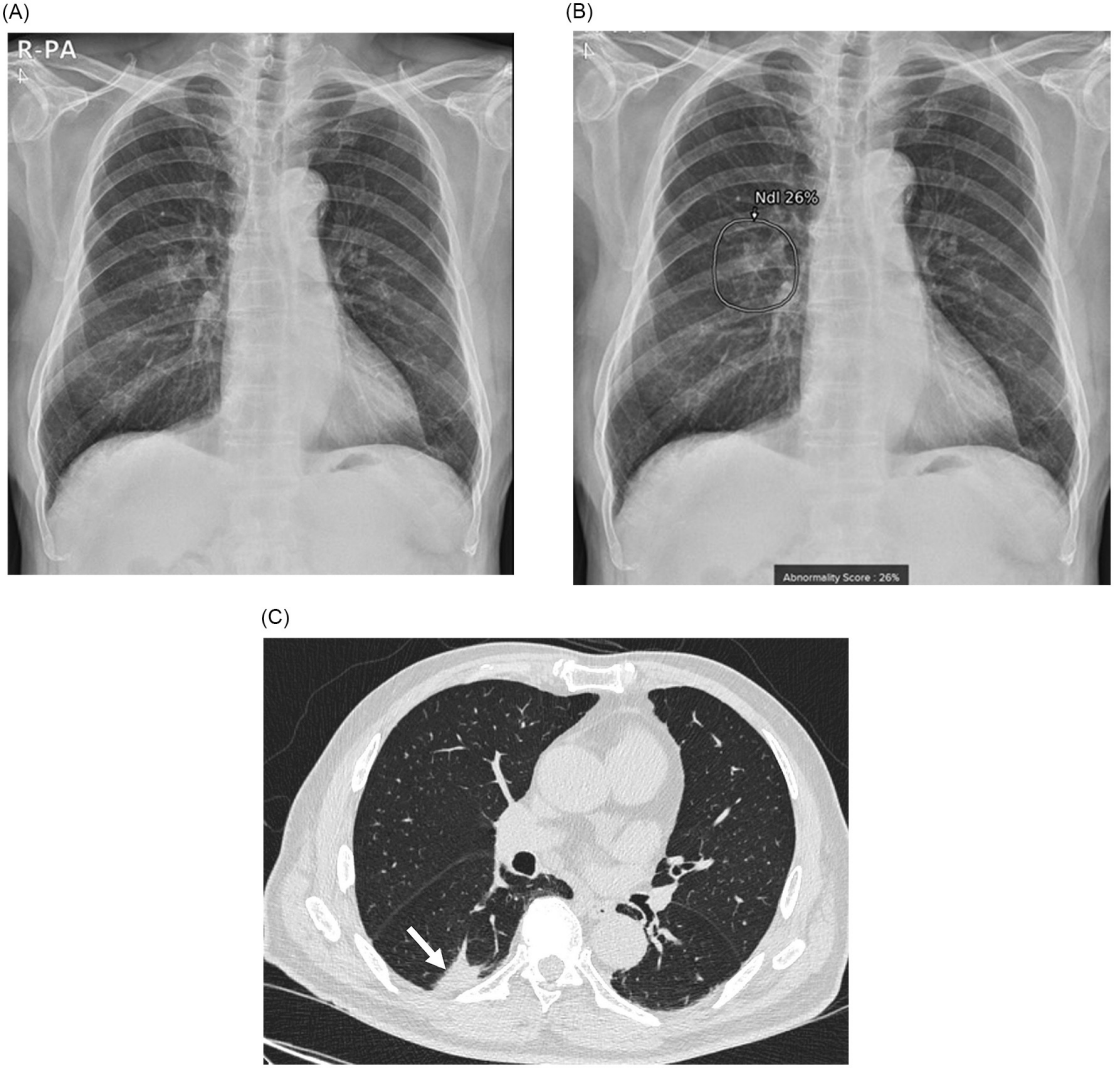
Chest radiograph and computed tomography (CT) of a 79-year-old man (#13 patient). (A) Chest radiograph was taken in the cardiomegaly outpatient clinic for the evaluation of arrhythmia. (B) Artificial intelligence-based lesion detection software detected a lung nodule in the right lower lobe with an abnormality score of 26%. (C) Chest CT demonstrated an irregular solid mass in the posterior subpleural space of the right lower lobe superior segment (arrow). After surgical resection, adenocarcinoma with an approximate size of 2.8 cm and the final stage of IA3 (pT1cN0M0) was confirmed.

After resection of the nodules, pathologic results confirmed the diagnosis of adenocarcinoma (n = 10, 76.9%), squamous cell carcinoma (n = 2), and small cell lung cancer (n = 1). Seven patients (53.8%) were in stage I, five patients were in stage II, and one was in stage III. According to the pathologic reports, the median size of the lesions was 2.6 cm (range, 1.9–5.5 cm). Most of the nodules were solid, but three lesions were part-solid (IA1 stage in #2 patient and IA3 stage in #1 and #8 patients). Total and invasive sizes of the part-solid nodules were 2.3–3.3 cm and 0.75–2.2 cm, respectively, and they were all detected as nodules by AI. Figs 2–4 showed chest radiograph, immediate AI-based results, and chest CT images of representative three lung cancer patients (Figs 2–4).

## Discussion

Among all patients with resectable lung cancer, 13 patients (17.3%) had incidentally detected lung cancer, and all of them had first undergone chest radiographs. Regardless of the size and characteristics of the nodules, AI-based lesion detection software detected all of the lesions as nodules with a median abnormality score of 78%. Eight patients underwent chest radiographs for the evaluation of extrapulmonary diseases, while the remaining five underwent radiographs in preparation of an operation or procedure concerning other body parts and were not expected to have pulmonary problems when they first underwent the chest radiographs. Even though they were not expected to have lung lesions, 61.5% of patients had a prompt consultation with a pulmonologist on the same day as the chest radiograph examination and before receiving the official radiologists’ report.

Seven patients (53.8%) were in stage I, which meant no additional treatment was required after surgery. This is clinically very important in terms of detecting early lung cancer, which could have been missed if the physicians had not paid attention to the screening chest radiographs, and it has great implications in that AI analysis could help prompt decision-making during this process. Chest radiograph is a basic examination that is performed countless times a day in an emergency room, outpatient clinic, preoperative, and inpatient screening examination even if there are no respiratory symptoms. However, in actual clinical practice, many doctors are implementing the real-time medical practice of reading the radiographs immediately before receiving the official radiologists’ report, and even if an abnormal finding is reported by the radiologists, it may be often overlooked or not considered in a timely manner. According to a previous study, about 20% of lung cancer patients had evidence of lung cancer in previous chest radiographs, which was missed at that time [24]. In the present study, 61.5% of patients had a prompt consultation with pulmonologists on the same day as the chest radiograph, suggesting that AI-based analysis in real-time could be valuable as a supporting tool for decision-making in clinical practice.

In the developing field of AI in radiology, chest radiograph is the most commonly adopted imaging modality for AI [25]. This is because chest radiograph is the most frequently performed imaging study, which results in a huge workload for radiologists who spend most of their working time on the interpretation of special studies such as ultrasonography, CT, or magnetic resonance imaging. However, chest radiograph is still a fundamental investigation for screening for diseases or preparing for treatment, and it has the power to guide the management of patients. Early and accurate diagnosis through chest radiograph is important because critical diseases, such as lung cancer or pneumothorax, can still be initially detected in chest radiographs. Lack of enough radiologists for prompt interpretation of chest radiographs at the outpatient clinic or emergency department may be a problem for clinicians who need to make urgent decisions based on chest radiographs. Therefore, AI has emerged as a novel solution as a computer-aided diagnosis system for chest radiographs. In our study, 8 patients had consultation before receiving the official radiologists’ report. Even though we could not compare the effect of official reports on patient management or survival due to the small number of the patients, this could be a reason why AI integration for chest radiographs has received more attention by researchers and doctors.

Especially for lung nodules, chest radiograph has limited sensitivity in the detection of lung cancer by radiologists, reported as 36–84% according to the nodule’s size and radiologist’s experience [26]. However, recent studies have examined the potential of utilizing AI for screening of lung nodules on chest radiographs and reported a sensitivity of 52–87% [26, 27]. According to them, radiologists or clinicians could have a better diagnostic performance when aided by AI for detecting malignant lung nodules [27–29]. Even though several studies have focused on developing and validating their own algorithm or commercially approved software for detecting lung nodules, most of them were retrospective studies simulating clinical situations [27, 28, 30, 31]. Clinical demand for validating AI in real-world practice is increasing nowadays, and according to a recent consensus statement, validation of AI for real-world clinical practice should be performed to confirm its usefulness [21]. Therefore, our study is meaningful because it showed how AI could actually play a useful role in the decision-making process of clinicians, especially in lung nodule detection tasks.

In our study, the median size of the resected lung cancers was 2.6 cm, and most of them were solid nodules. However, three cases were part-solid nodules, which are known to be difficult to detect in chest radiographs. In addition, one patient (#2 patient) had invasive lung adenocarcinoma of 0.75 cm size, which was also detected by the AI-based software. Further research on the effectiveness of AI for the detection of lung nodules and early lung cancer in actual clinical practice is necessary.

There are several limitations of our study. First, we did not analyze all chest radiographs that were interpreted to have a nodule by AI and did not review whether these nodules were actually proven to be lung cancer. Since this study evaluated cases with surgically resected lung cancer, it was difficult to demonstrate the diagnostic performance of AI for lung nodule detection and its usefulness for screening early lung cancer. In addition, cases of unexpectedly detected unresectable advanced lung cancer were not included in this study. Since it was not possible to compare before and after the introduction of AI, it was difficult to determine whether the introduction of AI actually increased the diagnosis rate of lung cancer and improved the patient’s prognosis. However, our study tried to highlight the usefulness of AI-based software for the detection of unexpected early lung cancer in a timely manner. Second, a controversy could exist regarding whether the clinicians referred to the radiologists’ reports or AI-based results at the time of consultation. We assumed that the clinicians were aided by the AI-based results immediately after the images were taken, based on the time recorded in the medical records. Whether the number or time of consultations was changed after AI usage could not be analyzed in this study. Nevertheless, our results are meaningful because they show how AI could aid in the decision-making process of clinicians, especially in lung cancer detection. Further prospective studies with large populations are needed to prove the clinical efficacy of AI.

## Conclusions

This study examined the actual cases of unexpectedly detected early lung cancer, which were all detected by AI-based lesion detection software as nodules. About 62% of the patients had a consultation with a pulmonologist for their abnormal chest radiograph findings on the same day when the radiograph was taken and before receiving the radiologists’ official report. With the increasing use of AI in medical imaging, the actual benefit of AI in chest radiographs for the detection of unexpected early lung cancer needs to be validated.

## Supporting information

S1 File Data file. (XLSX)

## Abbreviations

AI: artificial intelligence
CT: computed tomography
PACS: picture archiving and communicating system
